# Microcirculatory assessment of patients under VA-ECMO

**DOI:** 10.1186/s13054-016-1519-7

**Published:** 2016-10-25

**Authors:** Atila Kara, Sakir Akin, Dinis dos Reis Miranda, Ard Struijs, Kadir Caliskan, Robert J. van Thiel, Eric A. Dubois, Wouter de Wilde, Felix Zijlstra, Diederik Gommers, Can Ince

**Affiliations:** 1 Department of Intensive Care, Erasmus MC, University Medical Center Rotterdam, ‘s-Gravendijkwal 230, 3015 CE Rotterdam, The Netherlands; 2 Department of Cardiology, Erasmus MC, University Medical Center Rotterdam, ‘s-Gravendijkwal 230, 3015 CE Rotterdam, The Netherlands; 3 Department of Cardiothoracic Surgery, Erasmus MC, University Medical Center Rotterdam, ‘s-Gravendijkwal 230, 3015 CE Rotterdam, The Netherlands; 4 Department of Intensive Care, Hacettepe University Faculty of Medicine, Ankara, Turkey; 5 Department of Intensive Care, Erasmus MC, University Medical Center Rotterdam, Room H-603 ‘s-Gravendijkwal 230, 3015 CE Rotterdam, The Netherlands

**Keywords:** Microcirculation, VA-ECMO, Cardiogenic shock, Survival, ICU

## Abstract

**Background:**

Veno-arterial extracorporeal membrane oxygenation (VA-ECMO) is an effective technique for providing emergency mechanical circulatory support for patients with cardiogenic shock. VA-ECMO enables a rapid restoration of global systemic organ perfusion, but it has not been found to always show a parallel improvement in the microcirculation. We hypothesized in this study that the response of the microcirculation to the initiation of VA-ECMO might identify patients with increased chances of intensive care unit (ICU) survival.

**Methods:**

Twenty-four patients were included in this study. Sublingual microcirculation measurements were performed using the CytoCam-IDF (incident dark field) imaging device. Microcirculatory measurements were performed at baseline, after VA-ECMO insertion (T1), 48–72 h after initiation of VA-ECMO (T2), 5–6 days after (T3), 9–10 days after (T4), and within 24 h of VA-ECMO removal.

**Results:**

Of the 24 patients included in the study population, 15 survived and 9 died while on VA-ECMO. There was no significant difference between the systemic global hemodynamic variables at initiation of VA-ECMO between the survivors and non-survivors. There was, however, a significant difference in the microcirculatory parameters of both small and large vessels at all time points between the survivors and non-survivors. Perfused vessel density (PVD) at baseline (survivor versus non-survivor, 19.21 versus 13.78 mm/mm^2^, *p* = 0.001) was able to predict ICU survival on initiation of VA-ECMO; the area under the receiver operating characteristic curve (ROC) was 0.908 (95 % confidence interval 0.772–1.0).

**Conclusion:**

PVD of the sublingual microcirculation at initiation of VA-ECMO can be used to predict ICU mortality in patients with cardiogenic shock.

**Electronic supplementary material:**

The online version of this article (doi:10.1186/s13054-016-1519-7) contains supplementary material, which is available to authorized users.

## Background

Cardiogenic shock (CS) has a high mortality rate and is defined as a state of tissue hypoperfusion induced by cardiac failure [1, 2]. Many conditions, such as acute myocardial infarction [3], end-stage dilated cardiomyopathy [4], myocarditis [5], complications following cardiac surgery [6], and cardiac arrest [7], can cause CS. Veno-arterial extracorporeal membrane oxygenation (VA-ECMO) is an effective, portable, and rapidly deployable technique for providing emergency mechanical circulatory support for patients in CS [8–10], and it successfully reduces the time required for cardiac recovery [11]. Following this bridge therapy, some patients require implantation of a left ventricular-assist device (LVAD) or heart transplantation for adequate recovery and survival. Being able to identify hemodynamic variables related to survival could provide an important therapeutic window for optimizing VA-ECMO support. Until recently, lactate values have been suggested to predict outcomes of adult and pediatric patients on VA-ECMO [12–14]. However, these are downstream parameters and do not provide a direct hemodynamic endpoint.

Evaluation of systemic hemodynamic parameters, such as blood pressure, cardiac output, and mixed venous saturation, assist in diagnosing CS. VA-ECMO can quickly restore global hemodynamics. However, there is no guarantee that restoring these systemic hemodynamic variables will also improve microcirculatory and tissue perfusion, which is considered the ultimate success of resuscitation. Many studies have demonstrated that altered microcirculatory functional parameters can be a good predictor of mortality, despite normalized systemic hemodynamic variables [15–18]. The use of an intra-aortic balloon pump (IABP) did not affect the microcirculation parameters in these patients with or without VA-ECMO while restoring the macrocirculation [19, 20]. However, to date, no study has evaluated the sublingual microcirculatory response of patients with CS on VA-ECMO in relation to intensive care unit (ICU) outcomes after VA-ECMO. In this study, we tested the hypothesis that assessment of the microcirculation at initiation of VA-ECMO may identify patients with increased chances of ICU survival. To test this hypothesis we measured sublingual microcirculation in CS patients eligible for VA-ECMO therapy using hand-held microscopy.

## Methods

### Study setting and population

This study was conducted between September 2014 and October 2015 at the ICU of the Erasmus Medical Center Rotterdam, The Netherlands. All consecutive patients requiring VA-ECMO for CS were included in the study with the exception of two patients who died within 24 h for whom it was not possible to measure microcirculation due to continuous resuscitation in an overcrowded ICU box and for four patients who did not consent to inclusion in the study. Survival was defined as discharge from the ICU without a need for re-admission or re-implantation of the VA-ECMO after explantation. Additionally, bridged patients with durable LVADs or cardiac transplantations were documented as survivors. Non-survivors were those who died in the ICU during VA-ECMO support or within a couple of hours of explantation of VA-ECMO.

The following data were recorded at ICU admission: age, gender, body mass index, Sequential Organ Failure Assessment (SOFA) score [21], Acute Physiology and Chronic Health Evaluation (APACHE) II score, indications for VA-ECMO, heart rate, mean arterial pressure, lactate, hemoglobin (Hb), hematocrit (Htc), platelet count, lactate dehydrogenase (LDH), free hemoglobin (free-Hb), N-terminal of the prohormone brain natriuretic peptide (NT-proBNP), high sensitive troponin T (HsTnT), creatine kinase (CK), MB fraction of creatine kinase (CK-MB), and echocardiographic parameters.

### VA-ECMO circuit and placement, and patient management under VA-ECMO

VA-ECMO consisted of polyvinyl chloride tubing, a membrane oxygenator (Quadrox Bioline; Jostra-Maquet, Orleans, France), a centrifugal pump (Rotaflow; Jostra-Maquet), and either percutaneous arterial and venous femoral, or central right atrial and aortic cannulae (Biomedicus Carmeda; Medtronic, Boulogne-Billancourt, France). An oxygen-air blender (Sechrist Industries, Anaheim, CA, USA) ventilated the membrane oxygenator. Because of the percutaneous femoral VA-ECMO, an additional 7-F cannula was inserted distally into the femoral artery to prevent severe leg ischemia. Patients were kept on VA-ECMO for at least 48 h. The circuit was checked daily for significant fibrin deposition or clots accumulated on the membrane during hemolysis, thrombocythemia, or a sharp and systematic decline after blood oxygenation by experienced perfusionists and changed according to protocol.

### Microcirculatory measurements

Microcirculation measurements were performed using a CytoCam imaging device (CytoCam; Braedius Medical, Huizen, The Netherlands), which is a third-generation hand-held microscope [22]. The CytoCam device has a computer-controlled, high-resolution image sensor. Additionally, a CytoCam-IDF (incident dark field) device has a specifically designed microscopic lens that produces high-resolution images, showing approximately 30 % more capillaries than previous-generation devices [22–24]. The newly introduced lightweight (140 g versus previous devices weighing approximately 450 g) Cytocam-IDF device (with improved optics, a computer-controlled image sensor and illumination, and a more precise focusing mechanism) used in the present study allows for a faster and more stable and precise measurement to be made. In addition to these improvements, the device also has a faster measurement acquisition time, requiring only 3 to 5 s to assess the quality of the microcirculation. The sufficiency of this time was agreed upon in the second consensus conference held recently in November 2015 in Amsterdam and in March 2016 in Brussels, where most of the practitioners used the new Cytocam-IDF technology evaluated in the present study. Without applying pressure, the tip of the light guide is gently placed on the mucosal surface of the sublingual area. A 3- to 5-s video recording of predefined (left, right, and midline sublingual cavity) sites was obtained in each patient. Recordings were then blinded and analyzed to obtain the following microcirculatory parameters: total vessel density (TVD; mm/mm^2^), perfused vessel density (PVD; mm/mm^2^), proportion of perfused vessels (PPV; %), and microvascular flow index (MFI; arbitrary units (AU); a semiquantitative measure of microcirculatory flow [25]). Microcirculatory measurements were repeated at the following time points: baseline, after VA-ECMO insertion within 24 h (T1), 2–3 days after initiation of VA-ECMO (T2), 5–6 days after initiation of VA-ECMO (T3), 9–10 days after initiation of VA-ECMO (T4), and within 24 h of explantation of VA-ECMO. The timing between VA-ECMO insertion and the first microcirculatory and echocardiography measurements are shown in Table 1. The first echocardiography measurement was performed after a median of 9 (range 1–23) h and the microcirculatory measurement was performed after a median of 15 (range 1–22) h. Image clips were included which confirmed a quality score defined by Massey et al. [26]. Thus, 154 clips of 335 were excluded, and 181 clips were analyzed. Details of the excluded and included clips and the number of patients at each time point are shown in Additional file 1: Table S5. The most important reason for the poor quality of the clips was patient-related clinical conditions (such as bleedings, saliva, difficulty opening the mouth, difficulty inserting the device appropriately due to an endotracheal or gastric tube, and the lack of patient cooperation), especially in explanted patients. In addition, device-related conditions such as difficulties with focusing, brightness, and pressure artefacts resulted in poor-quality clips. Image clips were randomized and blinded to the three investigators (AK, SA, WdW) who performed the analysis using software to calculate the various functional microcirculatory parameters [27]. Microcirculatory parameters were divided into two categories: small vessels, defined as having a diameter of 25 μm or less (for example, TVD small vessel, PVD small vessel); and all vessels, defined as having small vessels and large vessels (>25–100 μm diameter; TVD all vessel, PVD all vessel) according to the consensus on microcirculatory assessment by De Backer et al. [25].

**Table 1:** Patient baseline characteristics

Demographics	Total (*n* = 24)	Survivors (*n* = 15)	Non-survivors (*n* = 9)	*P* value^a^
Age (years)	56 (20–70)	51 (23–70)	51 (20–67)	0.244
Male gender (*n* (%))	16 (67)	12 (80)	4 (44)	**0.08**
BMI (kg/m^2^)	24.5 (19–37)	25.0 (19–37)	24.0 (20–37)	0.904
SOFA score on admission day	11 (2–20)	10 (4–20)	12 (2–15)	0.309
APACHE II	32 (19–45)	31 (19–45)	34 (29–44)	0.244
VA-ECMO indications				
Dilated cardiomyopathy	1	0	1	ns
Ischemic cardiomyopathy	2	1	1	ns
Fulminant myocarditis	1	0	1	ns
Intoxication	1	1	0	ns
Pulmonary embolism	1	1	1	ns
Drowning	1	1	0	ns
Post-cardiectomy	5	4	1	ns
Cardiac arrest (*n* (%))	12 (50)	8	4	0.680
IHCA	4	3	1	
OHCA	8	5	3	
Global hemodynamics at admission				
Heart rate (beats/min)	93 (50–185)	91 (50–128)	95 (64–185)	0.858
MAP (mmHg)	67 (15–103)	71 (49–98)	66 (15–103)	0.269
Lactate (mmol/L)	4.1 (1.1–26)	4.0 (1.20–26)	6.2 (1.1–18)	0.743
Hb (mmol/L)	6.0 (4.7–11.3)	6.2 (4.7–11.30)	5.7 (5.0–7.9)	0.152
Htc (L/L)	0.32 (0.23–0.50)	0.30 (0.27–0.50)	0.28 (0.23–0.39)	0.100
Platelet count (1000/mm^3^)	136 (18–336)	136 (64–336)	148 (18–227)	0.835
LDH (U/L)	888 (264–16,779)	810 (264–16,779)	973 (360–3863)	0.493
Free-Hb (mg/dl)	4 (1–640)	4 (1.0–640)	4 (1.0–17)	0.516
HsTnT (ng/L)	4657 (45–89,641)	1683 (45–42,813)	13,369 (215–89,641)	**0.030**
CK (U/L)	2771 (27–18,526)	1082 (223–7286)	2637 (27–18,526)	0.114
CKMB (μg/L)	45.8 (1.2–859)	36.9 (2.5–412.1)	226 (1.2–859)	0.233
VA-ECMO flow (L/min)	4.14 (2.40–6.30)	4.0 (2.40–6.30)	4.1 (3.30–4.70)	0.929
Timing between VA-ECMO insertion and first microcirculation measurement (h)	15 (1–22)	15 (2–22)	13 (1–21)	0.857
Timing between VA-ECMO insertion and first echocardiography measurement (h)	9 (1–23)	7 (1–23)	11 (3–21)	0.426
Echocardiographic parameter at first 24 h after VA-ECMO implantation				
Aortic VTI (cm)	7 (5–22)	8,5 (5–22)	6 (5–12)	0.220
LVEF (%)	15 (7–55)	20 (8–55)	10 (7–20)	**0.010**
TDSa^b^ (cm/s)	5 (5–7)	5 (5–7)	5.5 (5–6)	1
TAPSE (cm)	10 (8–20)	10 (8–15)	8 (8–20)	0.845
Global hemodynamic parameters at first 24 h after VA-ECMO implantation				
CO^c^ (L/min)	2.1 (0.75–5.73)	2.46 (0.75–5.73)	1.37 (0.97–4.0)	**0.035**
CI^d^ (L/min/m^2^)	1.1 (0.39–2.81)	1.23 (0.39–2.81)	0.74 (0.48–1.69)	0.069
DO_2_^e^ (ml O_2_/min)	289 (122–790)	327 (150–790)	174 (122–510)	**0.006**
Fluid balance	1.35 (–1.90 to 4.00)	1.50 (–1.90 to 3.80)	1.0 (–1.10 to 4.0)	0.590
CVP (mmHg)	12 (1–32)	12 (1–32)	12 (5–17)	0.726
Microcirculation at first 24 h after VA-ECMO implantation				
All				
TVD (mm/mm^2^)	17.13 (11.88–30.39)	20.07 (15.71–30.39)	14.92 (11.88–23.99)	**0.008**
PVD (mm/mm^2^)	16.32 (11.01–29.60)	19.21 (12.99–29.60)	13.78 (11.01–18.47)	**0.001**
PPV (%)	96.48 (76.99–100)	98.06 (76.99–100)	89.20 (77–100)	**0.044**
MFI (AU)	2.97 (1.75–3.0)	3.0 (2.50–3.0)	2.87 (1.75–3.0)	0.191
Small				
TVD (mm/mm^2^)	15.70 (10.91–29.62)	18.85 (12.80–29.62)	11.84 (10.91–20.32)	**0.009**
PVD (mm/mm^2^)	14.16 (9.80–28.23)	18.61 (9.80–28.83)	11.01 (10.13–16.89)	**0.003**
PPV (%)	96.45 (43.59–100)	97.97 (76.46–100)	89.77 (82.93–100)	**0.038**
MFI (AU)	3 (0.75–3.0)	3.0 (2.25–3.0)	3.0 (0.75–3.0)	0.084
Outcome				
Total days on VA-ECMO (days)	5.5 (2–36)	6 (2–21)	5 (2–36)	0.588
ICU length of stay (days)	13.5 (2–65)	18 (6–65)	5 (2–36)	**0.03**
VA-ECMO-free days on ICU (days)	2.5 (0–52)	10 (0–52)	0	**0.0000**
Hospital length of stay (days)	17 (2–72)	22 (6–72)	5 (2–36)	**0.01**
ICU mortality (*n* (%))	9 (37.5)	0	9	
Hospital mortality (*n* (%))	11 (45.8)	2	9	

Categorical variables are presented as frequencies and percentages (*n* (%)) where stated; all other (continuous) variables are presented as median (range)

*APACHE* Acute Physiology and Chronic Health Evaluation, *AU* arbitrary units, *BMI* body mass index, *BSA* body surface area, *CI* cardiac index, *CK* creatine kinase, *CK-MB* myoglobin fraction of creatine kinase, *CO* cardiac output, *CVP* central venous pressure, *DO*_*2*_ oxygen delivery, *free-Hb* free hemoglobin, *Hb* hemoglobin, *HsTnT* high sensitive troponin T, *Htc* haematocrit, *ICU* intensive care unit, *IHCA* in-hospital cardiac arrest, *LDH* lactate dehydrogenase, *LVEF* left ventricular ejection fraction, *LVOT* left ventricular outflow tract, *MAP* mean arterial pressure, *MFI* microvascular flow index, *OHCA* out-of-hospital cardiac arrest, *PaO*_*2*_ arterial oxygen tension, *PPV* portion of perfused vessels, *PVD* perfused vessel density, *SaO*_*2*_ arterial oxygen saturation, *SOFA* Sequential Organ Failure Assessment, *TAPSE* Tricuspid annular plane systolic excursion, *TDSa* spectral tissue Doppler imaging mitral annulus peak systolic velocity, *TVD* total vessel density, *VA-ECMO* veno-arterial extracorporeal membrane oxygenation, *VTI* velocity time integral

^a^A Mann Whitney *U* test was used for comparison between the survivor and non-survivor groups; significant results are shown in bold text

^b^TDSa was only retrospectively found in 7 of the 24 patients’ echocardiography

^c^CO = heart rate × stroke volume, where stroke volume = LVOT area × LVOT VTI, and LVOT area was calculated by two-dimensional echocardiography using the following formula: (*π* × (LVOT diameter/2)2 (45)

^d^CI = CO/BSA, where BSA (in m^2^) was calculated according to the formula (by DuBois and DuBois) BSA = 0.20247 × height (m)^0.725^ × weight (kg)^0.425^

^e^DO_2_ = CO × CaO_2_ × 10, where CaO_2_ = (Hb × 1.34 × SaO_2_) + (PaO_2_ × 0.003); 0.003 is the solubility coefficient of oxygen in human plasma; each gram of hemoglobin is capable of carrying 1.34 mL of oxygen, and the amount of oxygen carried on the haemoglobin is Hb × 1.34 × SaO_2_

### Statistical analyses

Categorical variables are presented as frequencies and percentages. Continuous variables are presented as medians and range. Continuous variables were compared using a Mann–Whitney *U* test. For comparisons of microcirculatory parameters of patients in the same group at consecutive time points, a Friedman test was used. Baseline PVDs of all vessels, lactate HsTnT, and left ventricular ejection fraction (LVEF) were assessed as prognostic tests of survival using receiver operating characteristic curves (ROC) and their corresponding area under the curve. Cut-off points were calculated by obtaining the best Youden index (sensitivity + specificity – 1). Sensitivity, specificity, and positive and negative predictive values were calculated and compared to each other. With the cut-off values, an association between microcirculatory impairment and survival was explored. Statistical significance was defined as *p* < 0.05. Statistical analyses were performed using SPSS version 21.0.0.1 (SPSS, IBM, Armonk, NY, USA).

## Results

Thirty consecutive patients requiring VA-ECMO for CS were recruited to the study. Six patients were excluded: two patients who died within 24 h for whom it was not possible to measure microcirculation due to continuous resuscitation in an overcrowded ICU box, and four patients who did not consent to inclusion in the study. In total, 24 patients were included in the study.

Of the 24 patients who comprised the study population, 15 survived and 9 died on VA-ECMO. Eight patients were also inserted with an IABP in addition to VA-ECMO (two patients in the survival group (IABP was placed before ECMO), and six patients in the non-survival group received IABP (4 patients before ECMO and 2 patients during ECMO support)). The baseline characteristics, baseline global hemodynamics, and microcirculatory parameters are shown in Table 1. The median ages of the patients in the survivor and non-survivor groups were 51 (range 23–70) and 51 (20–67), respectively. The number of males was higher in the survivor group (*n* = 12; 80 %) compared to the non-survivor group (*n* = 4; 44 %). The APACHE II scores (31 (19–45) and 34 (29–44), respectively), SOFA scores (10 (4–20) and 12 (2–15), respectively), and systemic hemodynamics were not significantly different between the groups. The use of vasopressors and inotropic drug doses and blood transfusions did not statistically differ between groups on the first day of VA-ECMO or during VA-ECMO (Additional file 2: Table S6). Systemic hemodynamic variables, such as the lactate and hemoglobin values at the microcirculatory measurement time points, did not differ between survivors and non-survivors (Additional file 2: Table S6 and Additional file 3: cardiac output ROC, curve 5). While the cardiac output at baseline was statistically significantly different between the groups, the predictive value for ICU survival is less significant than the PVD measured sublingually. The cardiac function assessed by echocardiography was comparable between the groups, except for the LVEF 20 (8–55) and 10 (7–20); *p* = 0.010). The heart function biomarkers also showed no differences between the survivors and non-survivors. However, the baseline troponin levels at the initiation of VA-ECMO (1683 (45–42,813) ng/L and 13,369 (215–89,641) ng/L; *p* = 0.030) were significantly higher in the non-survivors. The ICU and hospital durations of stay were significantly different between the groups (18 (6–65) and 5 (2–36); and 22 (6–72) and 5 (2–36) days for survivors and non-survivors, respectively). The total number of days on VA-ECMO was similar for the survivor and non-survivor groups.

**Fig. 1:**
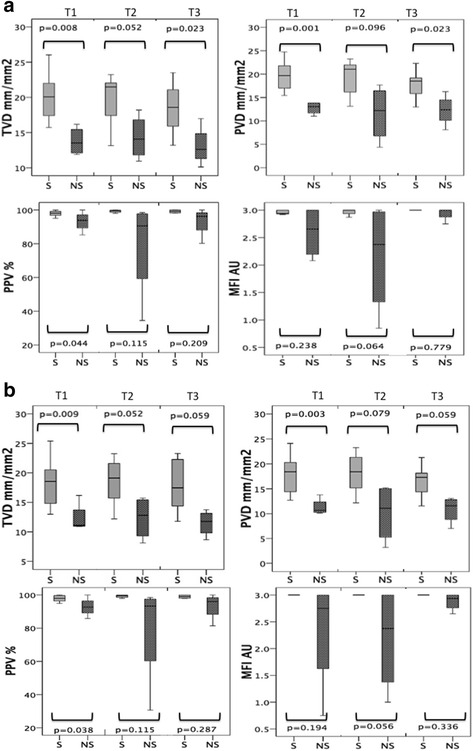
**a** Microcirculatory measurements showing the median and interquartile range between the survivors (*S*) and non-survivors (*NS*) for **a** all vessels and **b** small vessels at the initiation of the VA-ECMO insertion (*T1*), 48–72 h after VA-ECMO initiation (*T2*), and 5–6 days after VA-ECMO initiation (*T3*). *AU* arbitrary units, *MFI* microvascular flow index, *PPV* proportion of perfused vessels, *PVD* perfused vessel density, *TVD* total vessel density

**Fig. 2:**
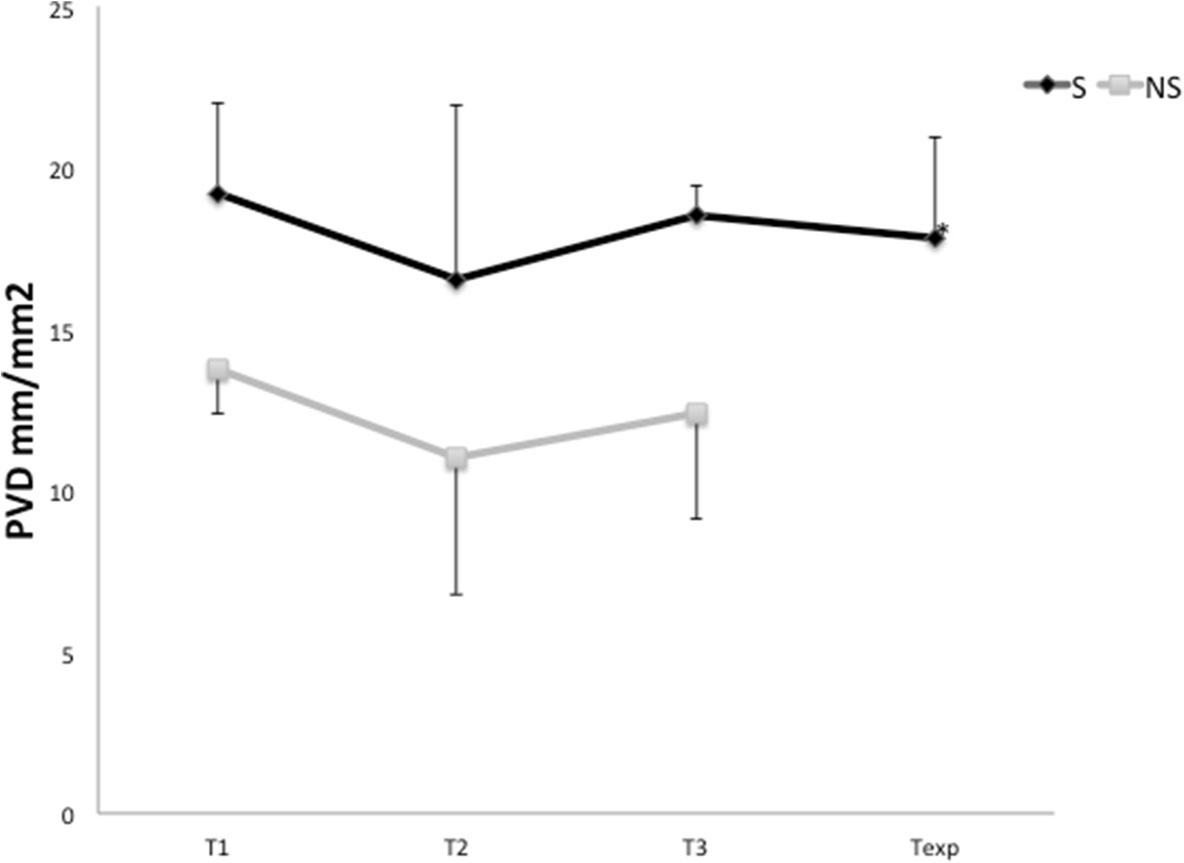
Example of change in the perfused vessel density (*PVD*) parameters in the survivor (*S*) and non-survivor (*NS*) groups at the following time points: initiation of the VA-ECMO insertion (*T1*), 48–72 h after VA-ECMO initiation (*T2*), 5–6 days after VA-ECMO initiation (*T3*), and after VA-ECMO explantation (*Texp*). The medians are depicted

**Fig. 3:**
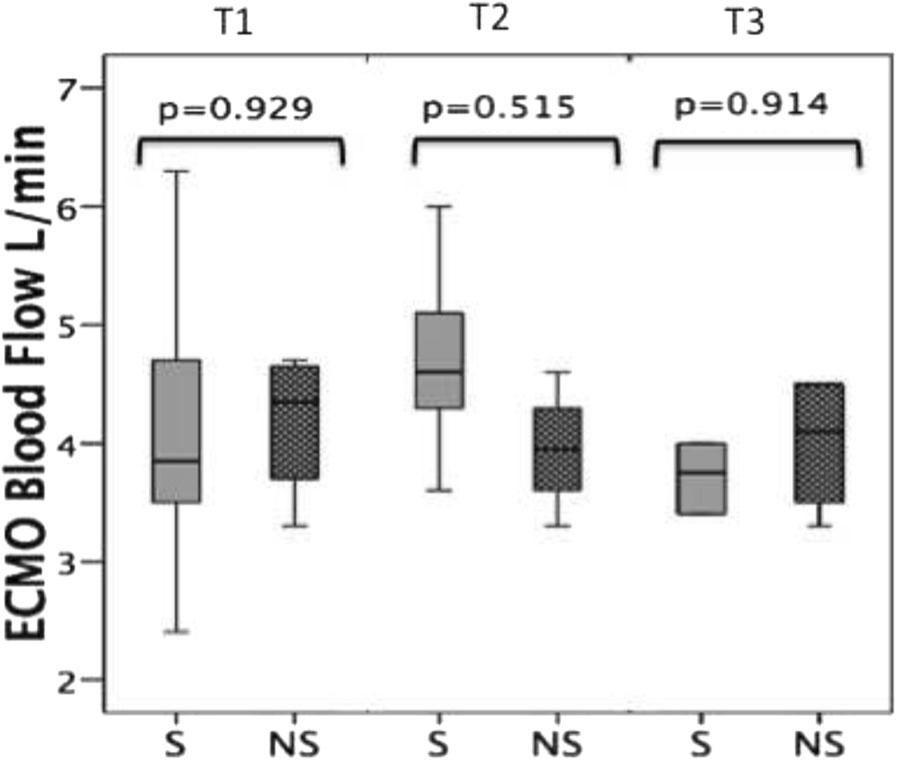
VA-ECMO blood flow showing the median and interquartile range between the survivors (*S*) and non-survivors (*NS*) at the initiation of the VA-ECMO insertion (*T1*), 48–72 h after VA-ECMO initiation (*T2*), and 5–6 days after VA-ECMO initiation (*T3*). *ECMO* extracorporeal membrane oxygenation

As shown in Fig. 1, the microcirculatory parameters at the initiation of VA-ECMO were significantly lower in the patients who subsequently did not survive compared to those who survived. The comparisons of measured microcirculatory parameters are shown for all time points in Additional file 4: Table S2. The microcirculatory parameters at T1 (i.e., the TVD, PVD, and PPV values) were significantly different between the survivor and non-survivor groups (all vessels *p* = 0.008, *p* = 0.001, and *p* = 0.044, respectively; small vessels *p* = 0.009, *p* = 0.003, and *p* = 0.038, respectively). The microcirculatory values decreased in both groups 3 days after the initiation of VA-ECMO at T2 in parallel with a reduction in arterial Hb, which is associated with hemodilution. Although the microcirculatory parameters of the non-survivor group somewhat recovered at day 6 (T3), they remained significantly lower than those in the survivor group (Fig. 2; Additional file 5: Table S3). All the non-survivors passed away while on VA-ECMO, whereas additional microcirculation was observed in the survivors following weaning from VA-ECMO. The microcirculatory parameters of the survivors then became slightly lower upon removal of VA-ECMO compared to their values while still on VA-ECMO (Fig. 2). Notably, the microcirculatory parameter values did not significantly change in time when on VA-ECMO. This effect was observed in both the survivors and non-survivors (Additional file 5: Table S3) and is shown in Fig. 3, where no difference in the VA-ECMO blood flow was observed between the initiation of VA-ECMO and all the other time points between the both groups. None of the surviving patients were bridged to a cardiac transplantation or LVAD in this period.

**Fig. 4:**
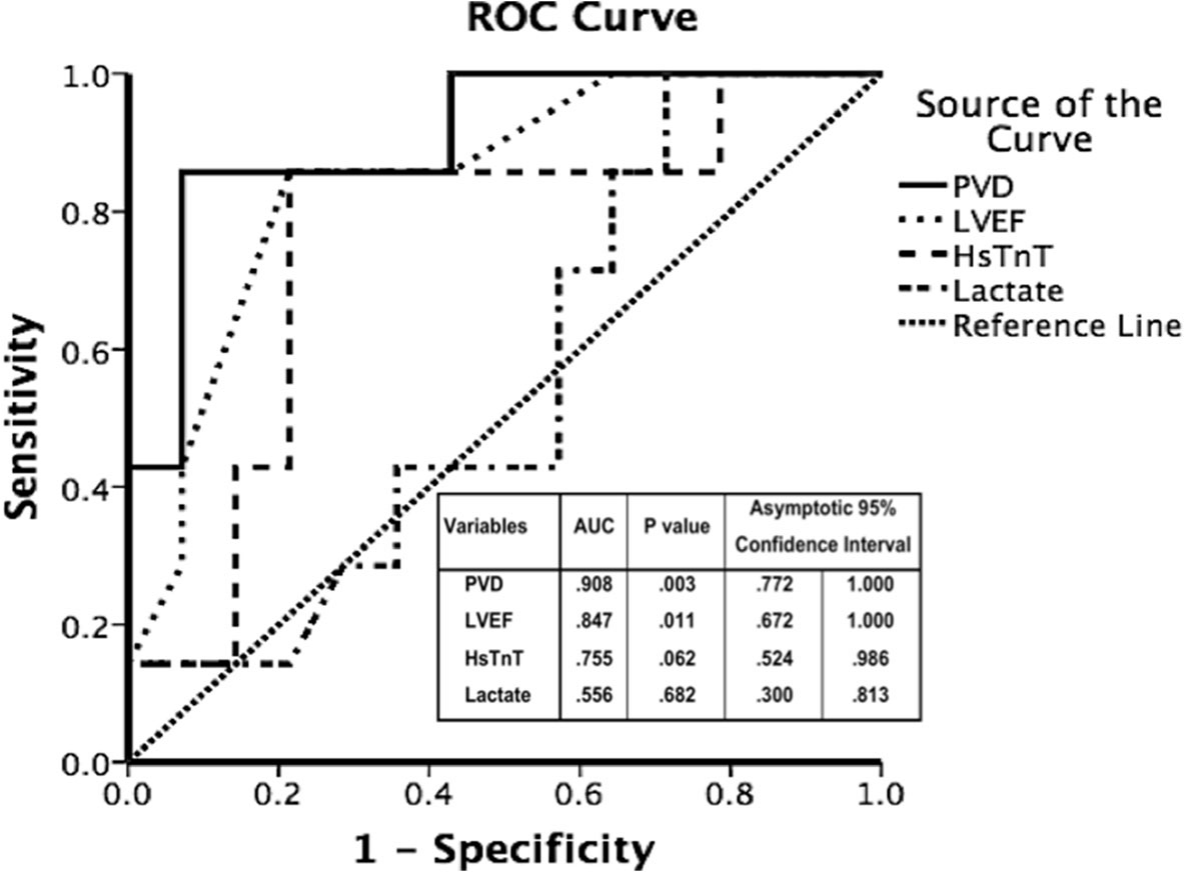
Receiver operating characteristics (*ROC*) curves showing the relationship between sensitivity and 1 – specificity in determining the perfused vessel density (*PVD*) all vessels, high sensitive troponin T (*HsTnT*), lactate, and left ventricular ejection fraction (*LVEF*) to predict ICU survival


To compare the sensitivity and specificity of the microcirculatory parameters for PVD in all vessels, LVEF, HsTnT, and lactate at the moment of initiation of VA-ECMO and at T1 were evaluated with ROC curve analysis to differentiate these values between the survivors and non-survivors (Fig. 4). The areas under the ROC curves (and the 95 % confidence interval) were 0.908 (0.772–1.0), 0.847 (0.672–1.0), 0.755 (0.524–0.986), and 0.556 (0.300–0.813) in the survivors and non-survivors, respectively. According to the ROC curve analysis, the threshold value of PVD for all the vessels was 15.2 (mm/mm^2^) and was found to predict ICU survival (sensitivity 88.9 %, specificity 86.7 %, positive predictive value 80 %, negative predictive value 92.9 %, and Youden index 0.75; Additional file 6: Table S4, Additional file 7, Additional file 8).

Additional file 7
**Clip 1** A surviving patient’s microcirculatory image clip recorded at the initiation of the VA-ECMO insertion (T1). (MOV 4513 kb)



Additional file 8
**Clip 2** A non-surviving patient’s microcirculatory image clip recorded at the initiation of the VA-ECMO insertion (T1). (MOV 3850 kb)


## Discussion

This prospective, observational, single-center study on patients with cardiogenic shock requiring VA-ECMO circulatory support showed that ICU survival could be predicted by observation of poor microcirculatory function at the initiation of VA-ECMO. Our study furthermore showed that VA-ECMO in itself did not change the physiological condition of the microcirculation throughout the course of VA-ECMO. These results suggest that assessment of sublingual microcirculation at initiation of VA-ECMO can be used to predict survival. While most of the differences are observed at baseline, changes over time seem less relevant. This seems to reflect more what occurred before, rather than during, ECMO. It also characterizes VA-ECMO as a bridge-to-treat procedure not contributing to resolving cardiac dysfunction following CS. From these considerations it could be argued that our results lay the foundation for introducing new adjunct therapeutic modalities aimed at improving microcirculatory function during the course of VA-ECMO, which may improve the chances of successful weaning from VA-ECMO and survival. Finally, our study suggests that microcirculatory monitoring could provide an easy tool to help the intensivist make the clinical decision to initiate or escalate further treatment options in patients with cardiogenic shock.

To date, several studies have evaluated predictors of outcome at the time of VA-ECMO initiation for CS. Schmidt et al. [28] described the value of pre-VA-ECMO implantation factors for predicting survival in cardiogenic shock patients receiving VA-ECMO on which they formulated a survival after VA-ECMO (SAVE) score. They found that this SAVE score could predict survival in these patients. Van Genderen et al. [29] investigated sublingual microcirculatory and peripheral tissue perfusion parameters in relation to systemic hemodynamics during and after therapeutic hypothermia following out-of-hospital cardiac arrest. They found that microcirculatory parameters were significantly lower in non-survivors at hospital admission and after rewarming than survivors. Microcirculatory alterations were also associated with organ failure and death, independent of changes in systemic hemodynamic parameters [29]. Aissaoui et al*.* [30] investigated predictors of successful VA-ECMO weaning after assistance for refractory CS. They described a number of echo criteria predictive for successful weaning. However, their study did not describe any predictors for patient survival. In this study, we found that baseline perfused vessel density is the best microcirculatory parameter predictive of ICU survival. Observation of low PVD measurements at initiation of VA-ECMO could lead to earlier initiation of advanced therapies such as LVAD or heart transplantations.

Another study we performed in pediatric ECMO patients [31] showed that patients who responded to ECMO with improved microcirculation went on to survive, whereas those that did not failed to survive.

A surrogate biomarker for left ventricular function was measured in our study. HsTnT was also found to be a good predictor for survival in the ROC analysis we performed. The study by Luyt et al. [32] assessed the predicative value of biomarkers for the prediction of cardiac recovery in patients on VA-ECMO who had not been investigated for HsTnT. However, their study investigated troponin Ic, which may have been the reason why they had found no relationship between cardiac biomarkers and cardiac recovery in VA-ECMO. Apart from HsTnT, LVEF was found to be the second best predictor for survival in the ROC analysis we performed. However, after initiating VA-ECMO, the heart is artificially bypassed and the cardiac output and ejection fraction are influenced by VA-ECMO. Thus, the echocardiography is affected by several factors such as aortic valve regurgitation, the aortic valve opening ratio, afterload change, and the VA-ECMO flow. In addition, transthoracic echocardiography is poor in quality, and transesophageal echocardiography is a less reliable ejection fraction measurement during VA-ECMO, which affects the measurement of the LVEF. However, even though the above parameters were related to the outcome to various degrees, our ROC analysis identified microcirculatory alterations measured on day 1 as having the highest specificity and sensitivity of all parameters measured for predicting mortality in the ICU.

In our observational study, volume status was mainly assessed by echocardiography and general hemodynamic parameters, such as the central venous pressure (CVP). We found no statistical significance between the two groups regarding the associations between fluid balance and CVP in the first 24 h and the outcome. It is worth pointing out, however, that even though the assessment of volume in non-VA-ECMO critically ill patients has extensively been studied and included in guidelines, accurate assessments of volume status in patients on ECMO with altered circulatory conditions (e.g., the presence of extracorporeal circulation and by-passing different parts of the endogenous circulation) is a subject in need of further investigation.

Our findings highlight the fact that ECMO is not a therapy that improves patient outcomes but rather it is a bridge to treatment. Our findings therefore identify a mean of 5 days as a window for treatment. It also means that additional therapeutic modalities targeting the microcirculation need to be developed for inclusion in the ECMO procedure, which may then elevate ECMO from the therapeutic modality status as a bridge to a treatment.

In the course of VA-ECMO, systemic perfusion is switched to an extracorporeal circulation, the blood is exposed to non-biocompatible polymers that can activate blood cells and the coagulation system, and it changes from a pulsatile to a non-pulsatile flow [33, 34]. While IABP may provide a pulsatile flow, many studies have shown that this was of no benefit to the microcirculation [20, 35, 36]. All of these factors might have detrimental effects on the microcirculation and may explain why the microcirculatory parameters decreased from T1 to T2 in both groups. At the same time, even though the VA-ECMO flows were similar in both groups, microcirculatory parameters were absolutely different at all time points. This means that there was no hemodynamic coherence between macrohemodynamics and the microcirculation and that microcirculatory alterations manifest themselves independently of systemic hemodynamics [37]. Furthermore, differences in microcirculatory measurements between the groups were observed at all time points, while persistent microcirculatory derangement was observed in the non-survivor group. In addition, our study showed that microcirculation remains high after VA-ECMO explantation in the survivor group.

Our study, however, has some limitations. First, it was single-center study and concerned a relatively small number of patients. However, to the best of our knowledge, this is the largest series of patients investigated on this topic to date. Second, we included patients who presented with different kinds of VA-ECMO cardiac injury. Therefore, these patients may have had different microcirculation properties prior to presentation. Another limitation was the exclusion of patients dying within 24 h. Fourth, apart from global hemodynamics, VA-ECMO can adversely affect the microcirculation for many reasons such as hemodilution, hemolysis, coagulopathy, non-pulsatile flow, and hypothermia [38]. T1 measurements were performed within 24 h after the initiation of VA-ECMO, which can be considered a long time period.

The last, but still controversial, limitation is the length of recording sequences used in our study. The use of a new generation hand-held microscopy device may have advantages, but also may have disadvantages. The advantage of the Cytocam IDF camera used in this study is that images are able to observe more capillaries (30 %) than the previous-generation sidestream dark field (SDF) cameras due to improved optics [22, 24]. In combination with a larger field of view (1.55 × 1.16 mm = about three times larger than the field of view of previous devices) of the new generation camera, it could be argued that this camera provides more accurate information concerning microcirculation alterations then previous-generation SDF camera. On the other hand, default Cytocam measurements take between 3- and 5-s recordings which have been found to be of sufficient length in a large number of recent studies [22, 39–42]. A recent review on microcirculation measurements by Massey and Shapiro [43] also recommended that lengths of between 3 and 5 s are sufficient. However, this length may be considered too short when comparing it to video sequences of 20 s recommended for SDF devices in the consensus of 2006 [25], although this recommendation was based on opinion and has never been validated in the literature. Even for SDF images, it has suggested in a recent experimental study by Kildal et al. [44] in pigs that measurements up to 10 min provide extra information. The only way to establish the sufficient length of the video sequences is to demonstrate statistical significance between the groups, which we did for this study. Whether such a length is sufficient for other applications, however, has to be investigated.

## Conclusion

In our study, altered baseline perfused sublingual microcirculatory vessel density was found to be related to ICU survival in cardiogenic shock patients treated with VA-ECMO. To our knowledge, this is the first study to describe such an application in this group of patients.

We conclude that monitoring sublingual microcirculation could be used for earlier identification of patients who will survive, as well as for exploring the usefulness of earlier consideration of the utilization of advanced therapies. Our expectation is that such assessment may be of value in the early identification of patients at a high risk of death, or those with poor recovery, and to potentially develop microcirculatory-targeted recovery strategies. Further studies with a larger number of patients and the same etiology of CS are needed.

## Additional files

Additional file 1: Table S5. Numbers of video clips and numbers of patients included at each time point. (DOCX 16 kb)
Additional file 2: Table S6. The median values of administered drugs, blood transfusions, and laboratory parameters at VA-ECMO insertion within 24 h (T1), 2–3 days after initiation of VA-ECMO (T2), and 5–6 days after initiation of VA-ECMO (T3). (DOCX 15 kb)
Additional file 3: Cardiac output ROC curve 5. (TIFF 1521 kb)
Additional file 4: Table S2. Comparison of the measured microcirculatory parameters TVD all vessel, TVD small vessel, PVD all vessel, PVD small vessel, PPV all vessels, PPV small vessel, MFI all vessel, and MFI small vessel at the initiation of the VA-ECMO insertion (T1), 48–72 h after VA-ECMO initiation (T2), and 5–6 days after (T3) between the survivors and non-survivors. (DOCX 16 kb)
Additional file 5: Table S3. Changes in the microcirculatory parameters in the survivor and non-survivor groups at the following time points: initiation of the VA-ECMO insertion (T1); 48–72 h after VA-ECMO initiation (T2); and 5–6 days after (T3). (DOCX 15 kb)
Additional file 6: Table S4. Sensitivity, specificity, positive predictive value, and negative predictive value for PVD all vessels, HsTnT, and LVEF to predict the ICU survival. (DOCX 14 kb)

## Abbreviations

APACHE: Acute Physiology and Chronic Health Evaluation
AU: Arbitrary units
CK: Creatine kinase
CS: Cardiogenic shock
CVP: Central venous pressure
ECMO: extracorporeal membrane oxygenation
free-Hb: Free hemoglobin
Hb: Hemoglobin
HsTnT: High sensitive troponin T
Htc: Hematocrit
IABP: Intra-aortic balloon pump
ICU: Intensive care unit
IDF: Incident dark field
LDH: Lactate dehydrogenase
LVAD: Left ventricular-assist device
LVEF: Left ventricular ejection fraction
MFI: Microvascular flow index
PPV: Proportion of perfused vessels
PVD: Perfused vessel density
ROC: Receiver operating characteristic curve
SAVE: Survival after VA-ECMO
SDF: Sidestream dark field
SOFA: Sequential Organ Failure Assessment
TVD: Total vessel density
VA-ECMO: Veno-arterial extracorporeal membrane oxygenation
