# Supplementary material for: Microcirculatory assessment of patients under VA-ECMO
Source: Crit Care. 2016 Oct 25;20:344. doi: 10.1186/s13054-016-1519-7 (PMC5078964; doi:10.1186/s13054-016-1519-7)
Supplement: Additional file 1: Table S5. — Numbers of video clips and numbers of patients included at each time point. (DOCX 16 kb) [file 13054_2016_1519_MOESM1_ESM.docx]

**Additional file 1: Table S5:** Numbers of video clips and numbers of patients were included in each time point

| **Time point** | **T1** | | **T2** | | **T3** | | **T4** | | **TEXP** | |
| --- | --- | --- | --- | --- | --- | --- | --- | --- | --- | --- |
| **Number of videos included** | 79_(20-37-22)*_ | | 53_(17-23-13)_ | | 20_(5-9-6)_ | | 8_(2-4-2)_ | | 21_(6-11-4)_ | |
|  | 50^**^  _(13-23-14)*_ | 29^***^  _(9-12-8)*_ | 34^**^  _(11-14-9)*_ | 19^***^  _(5-9-5)*_ | 13^**^  _(4-6-3)*_ | 7^***^  _(2-3-2)*_ | 5^**^  _(1-3-1)*_ | 3^***^  _(0-3-0)*_ | 21^**^  _(7-9-5)*_ | 0^***^ |
| **Number of Videos excluded** | 52 | | 38 | | 20 | | 13 | | 31 | |
| **Number of Patients included** | 24 | | 17 | | 8 | | 3 | | 9 | |
| **Reason of different Number of Patients included each time points**  **Died**  **ECMO explanted**  **Poor quality in all videos** | 0  0  0 | | 2  4  1 | | 6  10  0 | | 7  13  1 | | 0  0  2 | |

* Note the number of clip values given in parenthesis refer to the location of the measurement where (left, middle and right)

** Survivor; ***Non survivor
