# Supplementary material for: Microcirculatory assessment of patients under VA-ECMO
Source: Crit Care. 2016 Oct 25;20:344. doi: 10.1186/s13054-016-1519-7 (PMC5078964; doi:10.1186/s13054-016-1519-7)
Supplement: Additional file 2: Table S6. — The median values of administered drugs, blood transfusions, and laboratory parameters at VA-ECMO insertion within 24 h (T1), 2–3 days after initiation of VA-ECMO (T2), and 5–6 days after initiation of VA-ECMO (T3). (DOCX 15 kb) [file 13054_2016_1519_MOESM2_ESM.docx]

**Additional file 2: Table S6:** The median values of patient’s were administrated drugs, blood transfusions and were performed laboratory parameters at VA-ECMO insertion within 24 hours (T1); 2-3 days after initiation of VA-ECMO (T2); 5–6 days after initiation of VA-ECMO (T3).

|  | Total [n=24] | Survivor [n=15] | Non-survivor [n=9] | P values |
| --- | --- | --- | --- | --- |
| Norepinephrin mcg/kg/min  First ECMO day  Cumulative doses on ECMO support | 0.19 [0-0.53]  0.79[0-1.5] | 0.031[0-0.53]  0.69[0-1.5] | 0.060[0-0.39]  0.79[0-1.2] | 0.238  0.725 |
| Dobutamin mcg/kg/min  First ECMO day  Cumulative doses on ECMO support | 0 [0-4.4]  4.1 [0-21] | 0 [0-4.4]  4.1 [0-21] | 0 [0-3.4]  4.0 [0-20] | 0.793  0.808 |
| Red Blood transfusions (unit/day)  T1  T2  T3 | 0 [0-14]  0 [0-6]  1 [0-3] | 0 [0-14]  0 [0-6]  1 [0-2] | 0 [0-12]  0 [0-4]  1 [0-3] | 0.886  0.947  0.802 |
| Hemoglobin (mmol/L)  T1  T2  T3 | 6.0 [4.7–11.3]  5.6 [4.1–9.1]  6.0 [5.0–9.1] | 6.2 [4.7-11.30]  5.8 [4.1–9.1]  5.9[5.0–9.1] | 5.7 [5.0–7.9]  5.5 [4.1–8.6]  6.0 [5.6–8.9] | 0.152  0.722  0.962 |
| Lactate (mmol/L)  T1  T2  T3 | 4.1[1.1–26]  1.5 [0.7-4.5]  1.5 [0.7-4.4] | 4.0 [1.20–26]  1.7 [0.7-4.5]  1.3 [0.7-4] | 6.2 [1.1–18]  1.4 [0.7-4.3]  1.5 [0.9-4.4] | 0.743  0.591  0.345 |
