# Supplementary figures and images for: Microcirculatory assessment of patients under VA-ECMO
Source: Crit Care. 2016 Oct 25;20:344. doi: 10.1186/s13054-016-1519-7 (PMC5078964; doi:10.1186/s13054-016-1519-7)

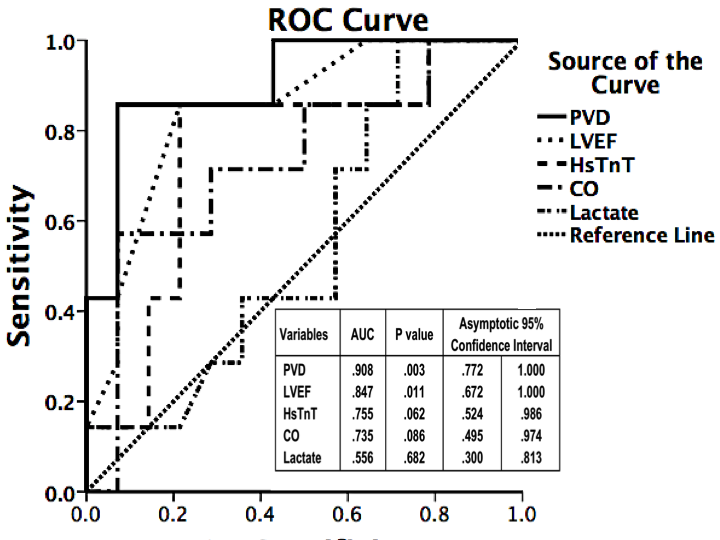

Supplement: Additional file 3: — Cardiac output ROC curve 5. (TIFF 1521 kb) [file 13054_2016_1519_MOESM3_ESM.tiff]
