# Supplementary material for: Microcirculatory assessment of patients under VA-ECMO
Source: Crit Care. 2016 Oct 25;20:344. doi: 10.1186/s13054-016-1519-7 (PMC5078964; doi:10.1186/s13054-016-1519-7)
Supplement: Additional file 4: Table S2. — Comparison of the measured microcirculatory parameters TVD all vessel, TVD small vessel, PVD all vessel, PVD small vessel, PPV all vessels, PPV small vessel, MFI all vessel, and MFI small vessel at the initiation of the VA-ECMO insertion (T1), 48–72 h after VA-ECMO initiation (T2), and 5–6 days after (T3) between the survivors and non-survivors. (DOCX 16 kb) [file 13054_2016_1519_MOESM4_ESM.docx]

**Additional file 4: Table 2** Comparison of the measured microcirculatory parameters: TVD all vessel, TVD small vessel, PVD all vessel, PVD small vessel, PPV all vessels, PPV small vessel, MFI all vessel and MFI small vessel at the initiation of the VA-ECMO insertion (T1); 48–72 h after VA-ECMO initiation (T2); 5–6 days after (T3) between the survivors and non-survivors.

| **Time point** | **Microcirculatory parameter** | **Survivor** | **Non-survivor** | **p value*** |
| --- | --- | --- | --- | --- |
| **T1** | **TVD All Vessel** | 20.07 | 14.92 | **0.008** |
|  | **TVD Small Vessel** | 18.90 | 11.84 | **0.009** |
|  | **PVD All Vessel** | 19.21 | 13.78 | **0.001** |
|  | **PVD Small Vessel** | 18.61 | 11.01 | **0.003** |
|  | **PPV All Vessel** | 98.06 | 89.20 | **0.044** |
|  | **PPV Small Vessel** | 97.97 | 89.77 | **0.038** |
|  | **MFI All Vessel** | 3.00 | 2.87 | 0.238 |
|  | **MFI Small Vessel** | 3.00 | 3.00 | 0.194 |
| **T2** | **TVD All Vessel** | 17.95 | 12.67 | 0.052 |
|  | **TVD Small Vessel** | 16.39 | 10.74 | 0.052 |
|  | **PVD All Vessel** | 16.53 | 11.00 | 0.096 |
|  | **PVD Small Vessel** | 15.55 | 9.42 | 0.079 |
|  | **PPV All Vessel** | 97.16 | 89.34 | 0.115 |
|  | **PPV Small Vessel** | 97.30 | 89.90 | 0.115 |
|  | **MFI All Vessel** | 3.00 | 2.00 | 0.064 |
|  | **MFI Small Vessel** | 3.00 | 2.00 | 0.056 |
| **T3** | **TVD All Vessel** | 18.58 | 12.60 | **0.023** |
|  | **TVD Small Vessel** | 17.46 | 11.75 | 0.059 |
|  | **PVD All Vessel** | 18.52 | 12.38 | **0.023** |
|  | **PVD Small Vessel** | 17.30 | 11.58 | 0.059 |
|  | **PPV All Vessel** | 99.16 | 96.20 | 0.209 |
|  | **PPV Small Vessel** | 99.11 | 96.03 | 0.287 |
|  | **MFI All Vessel** | 3.00 | 3.00 | 0.779 |
|  | **MFI Small Vessel** | 3.00 | 2.93 | 0.336 |

*Mann Whitney U test was used.
