# Supplementary material for: Microcirculatory assessment of patients under VA-ECMO
Source: Crit Care. 2016 Oct 25;20:344. doi: 10.1186/s13054-016-1519-7 (PMC5078964; doi:10.1186/s13054-016-1519-7)
Supplement: Additional file 5: Table S3. — Changes in the microcirculatory parameters in the survivor and non-survivor groups at the following time points: initiation of the VA-ECMO insertion (T1); 48–72 h after VA-ECMO initiation (T2); and 5–6 days after (T3). (DOCX 15 kb) [file 13054_2016_1519_MOESM5_ESM.docx]

**Additional file 5: Table 3** Change in the microcirculatory parameters in the survivor and non-survivor groups at the following time points: initiation of the VA-ECMO insertion (T1); 48–72 h after VA-ECMO initiation (T2); 5–6 days after (T3)

| **Patients** | **Microcirculatory parameters** | **Microcirculatory Measurement Time Points** | | | | **P value*** |
| --- | --- | --- | --- | --- | --- | --- |
|  |  | **T1** | **T2** | **T3** | **Texp** |  |
| **Survivor** | **TVD All Vessel** | 20.07 | 17.95 | 18.58 | 18.67 | 0.494 |
|  | **TVD Small Vessel** | 18.90 | 16.39 | 17.46 | 16.60 | 0.392 |
|  | **PVD All Vessel** | 19.21 | 16.53 | 18.52 | 17.82 | 0.392 |
|  | **PVD Small Vessel** | 18.61 | 15.55 | 17.30 | 16.31 | 0.284 |
|  | **PPV All Vessel** | 98.06 | 97.16 | 99.16 | 97.72 | 0.656 |
|  | **PPV Small Vessel** | 97.97 | 97.30 | 99.11 | 98.82 | 0.656 |
|  | **MFI All Vessel** | 3.00 | 3.00 | 3.00 | 3.00 | 0.956 |
|  | **MFI Small Vessel** | 3.00 | 3.00 | 3.00 | 3.00 | 0.706 |
| **Non-Survivor** | **TVD All Vessel** | 14.92 | 12.67 | 12.60 |  | 0.779 |
|  | **TVD Small Vessel** | 11.84 | 10.74 | 11.75 |  | 0.472 |
|  | **PVD All Vessel** | 13.78 | 11.00 | 12.38 |  | 1.00 |
|  | **PVD Small Vessel** | 11.01 | 9.42 | 11.58 |  | 1.00 |
|  | **PPV All Vessel** | 89.20 | 89.34 | 96.20 |  | 0.779 |
|  | **PPV Small Vessel** | 89.77 | 89.90 | 96.03 |  | 0.779 |
|  | **MFI All Vessel** | 2.87 | 2.00 | 3.00 |  | 0.526 |
|  | **MFI Small Vessel** | 3.00 | 2.00 | 2.93 |  | 0.735 |

*Friedman test was used.
