# Supplementary material for: Microcirculatory assessment of patients under VA-ECMO
Source: Crit Care. 2016 Oct 25;20:344. doi: 10.1186/s13054-016-1519-7 (PMC5078964; doi:10.1186/s13054-016-1519-7)
Supplement: Additional file 6: Table S4. — Sensitivity, specificity, positive predictive value, and negative predictive value for PVD all vessels, HsTnT, and LVEF to predict the ICU survival. (DOCX 14 kb) [file 13054_2016_1519_MOESM6_ESM.docx]

**Additional file 6: Table S4** Sensitivity, specificity, positive predictive value and negative predictive value showing the PVD all vessels, HsTnT to predict the ICU survival.

| **Limit Value** | **Sensitivity (%)** | **Specificity**  **(%)** | **Positive Predictive Value (%)** | **Negative Predictive Value (%)** |
| --- | --- | --- | --- | --- |
| **PVD All Vessel**  **(15.2 mm/mm^2^)** | **88.9** | **86.7** | **80** | **92.9** |
| **HsTnT**  **(>10.000 ng/l)** | **77.8** | **80** | **70** | **85.7** |
| **LVEF**  **(≤10)** | **85.7** | **78.6** | **77** | **83.3** |
